# Age based evaluation of nut aspiration risk

**DOI:** 10.1186/s40463-020-00473-y

**Published:** 2020-10-09

**Authors:** Jill N. D’Souza, Taher S. Valika, Bharat Bhushan, Jonathan B. Ida

**Affiliations:** 1 Division of Pediatric Otolaryngology – Head and Neck Surgery, Children’s Hospital New Orleans, Louisiana State University, New Orleans, LA USA; 2 Divison of Otolaryngology, Head & Neck Surgery, Ann & Robert H. Lurie Children’s Hospital of Chicago, Chicago, IL USA; 3 Northwestern University, Feinberg School of Medicine, Chicago, IL USA

**Keywords:** Pediatric, Airway, Foreign body, Bronchoscopy, Aspiration, Peanut

## Abstract

**Objective:**

To identify an age at which initiation of whole nut into the pediatric diet could be considered safe, by evaluating the age distribution of children undergoing bronchoscopy with removal of nut or seed material from the airway.

**Method:**

A retrospective chart review over a ten-year period identifying children age 0–18 that have undergone bronchoscopy with retrieval of airway foreign bodies. A statistical analysis of demographic data was carried out to identify age distribution of aspiration events.

**Results:**

Sixty-four cases of foreign body aspiration were identified, of which 43 (67%) were of organic origin, specifically nuts. A Fisher’s exact test was carried out on the cumulative percentage of organic foreign body aspirations to identify the age distribution of nut aspiration events. A statistically significant decrease in organic foreign body aspirations occurred at approximately 36 months of age (*p* = 0.004).

**Conclusion:**

Foreign body aspiration is a leading cause of accidental injury or death in children. Nut and other small organic foreign bodies account for a significant portion of accidental aspiration events, however, no guidelines exist regarding appropriate age of whole nut introduction into the diet. Our study suggests that 90% of pediatric nut aspiration events occur under the age of 36 months. We suggest supervised introduction of whole nuts between the ages of 3 and 4 years. Official guidelines regarding this should be considered by professional pediatric societies.

**Level of evidence:**

4

## Introduction

A 2017 New York Times article highlighted the importance of exposing children to peanuts in their diet “early and often;” however, it did not specify the fact that the recommended peanut exposure should be in the form of powder or paste [1]. Pediatric aspiration of airway foreign body is the admitting diagnosis to US hospitals for approximately 2000 children annually [2]. The mortality rate has been reported as ranging between 0.3 and 2.5% [2, 3]. While prior studies have data revealing age-based information about nut aspiration, there is no study in the literature focused specifically on examining age-based characteristics of nut aspiration [4–6]. In particular, there is no current evidence-based guidelines regarding introduction of whole nuts into the pediatric diet, but rather is left to parental discretion, without guidance from proper professional healthcare societies.

Airway foreign body aspiration is a major concern in the pediatric patient presenting with sudden onset coughing spells, respiratory distress, or choking. These patients are usually seen in the emergency department and undergo radiographic evaluation with chest x-ray or airway CT scan to evaluate presence of lung hyperinflation or air-trapping. However, even with negative or ambiguous radiographic data, many surgeons will proceed with rigid bronchoscopic evaluation based on age and a suspicious history [4]. Bronchoscopy is the gold standard of diagnosis for airway foreign body [5], however subjects the child to the risks of anesthesia.

The aim of this study was to examine nut foreign body aspiration and ascertain if there is an age at which risk of nut aspiration declines, thereby helping establish the groundwork for future dietary introduction guidelines.

## Materials and methods

This research proposal was approved by the Lurie Children’s Hospital Institutional Review Board (IRB 2017–957). A retrospective chart review of patients that underwent rigid bronchoscopy with airway foreign body retrieval over a 10-year period (2007–2017) was undertaken. Sampling was done by selecting for the *Current Procedural Terminology* code 31635, for Bronchoscopy, rigid or flexible, with or without fluoroscopic guidance, with removal of foreign body. Age at procedure, preoperative comorbidities, type of foreign body, location of foreign body, gender, and duration of procedure were retrieved and included in this study. Data was analyzed with SAS 9.3 (SAS Institute, Cary, NC) to compare specific study groups.

## Results

The search identified 66 patients who underwent rigid bronchoscopy with foreign body retrieval at Lurie Children’s Hospital between 2007 and 2017 via the aforementioned search methodology. Two patients were excluded after chart review revealed that the foreign body location was extraneous to the airway, leaving 64 patients in the study. There were 29 (45%) female and 35 (55%) male children in this group. Most children had no underlying comorbidities; other than a single child with prematurity, laryngomalacia, seizures, cardiac defects, asthma, and developmental delay. No preexisting diagnosis of dysphagia was noted in the study population. Racial classification included 5% African American, 19% Caucasian, 23% Hispanic, 36% who did not identify as Hispanic or Latino, and 17% in which the race was not defined.

Forty-four (69%) patients were found to have aspirated a nut, while 20 (31%) had an inorganic foreign body within the airway identified at bronchoscopy. Table 1 indicates average age (months) and gender distribution amongst children that underwent bronchoscopy for airway foreign body, and shows that average age at aspiration for whole nut was 24 months, while average age for inorganic material was older, at 74 months.

**Table 1:** Demographic data of airway foreign bodies

Demographic Data	Overall	Inorganic	Organic
Age at Aspiration (average months)	39.7	74.3	24.1
Gender	45% female	55% female	41% female

**Fig. 1:**
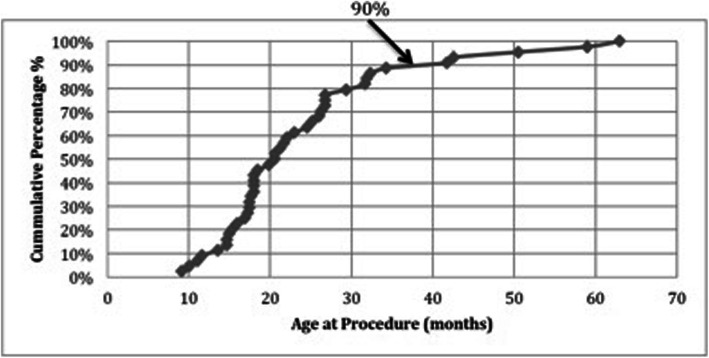
Age at retrieval of aspirated organic foreign body

Data was processed and statistically analyzed in order to determine at what age the frequency of nut aspiration declines. The age of patients was expressed in months. The number of nut aspiration events by patient with progression of age was calculated, and expressed using the cumulative percentage method. We found that 90% of organic foreign body aspirations occurred at approximately 36 months of age (Fig. 1). A Fisher’s exact test was then used to compare the cumulative frequency percentages of organic airway foreign body aspiration before and after 36 months of age, and found to be statistically significant (*p* = 0.004). Data was analyzed with SAS 9.3 (SAS Institute, Cary, NC) to compare specific study groups. Only three aspirations occurred after 4 years of age, and only one aspiration occurred after 5 years of age.

## Discussion

Aspiration of foreign bodies in the pediatric population is associated with significant morbidity, including exposure to radiation for imaging, and anesthetic exposure for diagnostic bronchoscopy with retrieval of the foreign body. In a 2010 article, the American Academy of Pediatrics released a policy statement on the importance of appropriately labeling choking hazards due to risk of aspiration-related injury or death [6]. Normal childhood development includes bringing hand to mouth starting as early as age 2 months, and children explore their environment frequently by placing unknown objects in their mouths [7]. In children under age 3, the airway is particularly at risk for aspiration as the protective chewing and swallow mechanisms have not fully developed. Dental development sequences also contribute to this, as the incisors develop around 8–13 months of age, with molars presenting around 16–29 months [8]. This allows young children to take larger bites of food materials without the corresponding ability to grind them into smaller pieces [7, 8], which in turn may increase the risk of food particle aspiration.

In 2000, the American Academy of Pediatrics recommended that foods with high allergy potential, such as eggs, peanuts, milk, corn, soy, and citrus, be avoided in high risk infants (infants from families with significant atopic history) for the first 2–4 years of life [9]. In recent years, however, there has been significant interest regarding early introduction of allergenic material. A UK-Israeli study compared the rates of peanut allergy between Israeli children raised in the UK versus children raised in Israel, and found the rate was much higher in the British cohort, likely due to earlier and more frequent consumption of peanut-based products in Israel [10, 11]. Specific attention should be paid to the rise in nut allergies, and the commendable efforts by the National Institute of Allergy to decrease the rate of peanut allergy by introducing peanut products at an early age. However, it is essential to use caution when introducing whole nuts and similar size and consistency organic material into the pediatric diet. Therefore, data regarding the age at which nut aspiration declines is essential in building information for potential recommendations on the introduction of whole nuts into the diet. Our data demonstrates a statistically significant decline in the rate of organic foreign body retrieved from the airway after age 3, and almost none over the age of 4. While the sample size is small (*n* = 64), we achieved sufficient power to determine a statistically significant distribution of the cumulative percentage of nut aspiration, with a plateau identified around age 36 months.

Prior studies investigating airway foreign bodies have revealed similar age-based data, despite varied conclusions. Data from Midulla, et al. determined that 90% of pediatric airway foreign bodies occurred in children under 3 years old, with a noted peak incidence in the second year [12]. This data represented the entirety of aspirated foreign bodies, and not isolated to nut-related material. Mu, et al. revealed similar findings in terms of the incidence of airway foreign body aspiration, though did not elaborate on nut-specific data [13]. Others have also identified that the likelihood of an aspirated foreign body being a whole nut is higher than an inorganic object [14], though the data did not investigate the ages at which this occurred.

In addition to anticipated airway age-appropriate developmental vulnerabilities, consideration should also be given to consequences of airway foreign body aspiration. Mortality has been estimated between 0.3 and 2.5%; however, no reliable assessment of morbidity exists. In particular, children who are suspected to have an airway foreign body typically undergo rigid bronchoscopy under general anesthesia in order to fully examine the airway. Standard chest x-ray is routinely completed, but may be equivocal in the presence of acute nut-related aspiration with some studies even revealing that two-thirds of the children with airway foreign bodies have normal x-ray findings [13, 15, 16]. While future diagnostic tools may include airway CT [17], it has not replaced direct surgical endoscopy as the gold standard to rule out an airway foreign body.

In April 2017, the FDA released new pediatric guidelines suggesting caution with the use of anesthesia in children under the age of 3, given the risk of harm to neurocognitive development [18]. Given these guidelines, every attempt should be made at minimizing situations in which a child under the age of 3 requires a surgical intervention. Organic foreign body aspiration occurs almost entirely (90%) under this age and may be minimized by careful attention to the pediatric diet and what is accessible to young children.

The 2017 New York Times article entitled *Feed Your Kids Peanuts, Early and Often, New Guidelines Urge* highlighted the importance of exposing children to peanuts in their diet at a young age [1]; however, the title and accompanying headline photograph suggested whole nuts be used, rather than the paste or powder recommended in the 2017 Guidelines for the Prevention of Peanut Allergy issued by the National Institute of Allergy and Infectious Disease [19]. Headlines and accompanying photos like this may lead parents to falsely assume whole nuts are safe for their children regardless of age. In fact, we suggest extreme caution should be used when introducing nuts or similar sized and consistency organic material to young children and avoiding it entirely in children under age 3. Given decrease in events over age 3 years, carefully supervised introduction of whole nuts may be considered between the ages of 3 and 4 years in the developmentally appropriate child. There is no age at which the probability of foreign body aspiration can be estimated to zero, as bronchoscopy for foreign body aspiration does take place in the adult population [20, 21]. These recommendations come with limitations as our data set contained only 44 children. It would be beneficial to investigate in a multi-center fashion, even though our data was statistically significant, to increase the overall power.

To our knowledge, this is the first paper to directly investigate the age distribution of whole nut aspiration, though there are sources in the pediatric literature that describe a greater incidence of all foreign body aspiration in children under age 3 [12, 13]. As care for the pediatric patient is so often centered around their primary care physician, recommendations from professional pediatric societies would help standardize introduction of whole nuts into the pediatric diet.

## Conclusion

This study demonstrated that 90% of whole nut aspiration occurs under 36 months of age, and almost none occur over the age of 4 years. In children under age 3 years, our data suggests that ingestion of whole nuts should be avoided entirely; between the ages of 3 and 4 years, careful supervised introduction may be considered in the developmentally appropriate child. With increasing awareness of nut allergy, and a well-intentioned desire to avoid these in children, it is essential to provide professional guidance as to the safety issues surrounding whole nut consumption in children, and strategies to provide age-appropriate nut exposure. It is our hope that this data may serve as a starting point to the development of national guidelines regarding introduction of whole nuts to the pediatric diet.

## Data Availability

The datasets during and/or analyzed during the current study available from the corresponding author on reasonable request.

## References

[1] Rabin RC. Feed Your Kids Peanuts, Early and Often, New Guidelines Urge. *The New York Times*. https://www.nytimes.com/2017/01/05/well/eat/feed-your-kids-peanuts-early-and-often-new-guidelines-urge.html. Published January 5, 2017. Accessed February 20, 2020.

[2] Kim IA, Shapiro N, Bhattacharyya N (2015). The national cost burden of bronchial foreign body aspiration in children. Laryngoscope..

[3] Roberts CA, Carr MM (2018). Morbidity and mortality in children undergoing bronchoscopy for foreign body removal. Laryngoscope..

[4] Cutrone C, Pedruzzi B, Tava G (2011). The complimentary role of diagnostic and therapeutic endoscopy in foreign body aspiration in children. Int J Pediatr Otorhinolaryngol.

[5] Sink JR, Kitsko DJ, Georg MW, Winger DG, Simons JP (2016). Predictors of foreign body aspiration in children. Otolaryngol Head Neck Surg.

[6] Committee on Injury, Violence, and Poison Prevention (2010). Prevention of choking among children. Pediatrics..

[7] Byard RW, Gallard V, Johnson A, Barbour J, Bonython-Wright B, Bonython-Wright D (1996). Safe feeding practices for infants and young children. J Paediatr Child Health.

[8] Nelson SJ, Ash MM, Ash MM. Wheeler’s dental anatomy, physiology, and occlusion. 9th ed: Saunders/Elsevier; 2010.

[9] American Academy of Pediatrics. Committee on Nutrition (2000). Hypoallergenic infant formulas. Pediatrics..

[10] Du Toit G, Katz Y, Sasieni P (2008). Early consumption of peanuts in infancy is associated with a low prevalence of peanut allergy. J Allergy Clin Immunol.

[11] Elizur A, Katz Y (2016). Timing of allergen exposure and the development of food allergy: treating before the horse is out of the barn. Curr Opin Allergy Clin Immunol.

[12] Midulla F, Guidi R, Barbato A (2005). Foreign body aspiration in children. Pediatr Int.

[13] Mu L, He P, Sun D (1991). Inhalation of foreign bodies in Chinese children: a review of 400 cases. Laryngoscope..

[14] Bakal Ü, Keleş E, Saraç M, Karlidağ T, Kaygusuz İ, Kazez A (2016). A study of foreign body aspiration in children. J Craniofac Surg.

[15] Brown JC, Chapman T, Klein EJ (2013). The utility of adding expiratory or decubitus chest radiographs to the radiographic evaluation of suspected pediatric airway foreign bodies. Ann Emerg Med.

[16] Zerella JT, Dimler M, McGill LC, Pippus KJ (1998). Foreign body aspiration in children: value of radiography and complications of bronchoscopy. J Pediatr Surg.

[17] Pitiot V, Grall M, Ploin D, Truy E, Ayari KS (2017). The use of CT-scan in foreign body aspiration in children: a 6 years’ experience. Int J Pediatr Otorhinolaryngol.

[18] Research C for DE and FDA Drug Safety Communication (2019). FDA approves label changes for use of general anesthetic and sedation drugs in young children.

[19] Togias A, Cooper SF, Acebal ML (2017). Addendum guidelines for the prevention of Peanut allergy in the United States: report of the National Institute of Allergy and Infectious Diseases-sponsored expert panel. J Pediatr Nurs.

[20] Hewlett JC, Rickman OB, Lentz RJ, Prakash UB, Maldonado F (2017). Foreign body aspiration in adult airway. J Thorac Dis.

[21] Sehgal IS, Dhooria S, Ram B (2015). Foreign body inhalation in the AdultPopulation: experience of 25,998 Bronchosccopis and systematic review of the literature. Respir Care.

